# Supplementary figures and images for: Constitutive upregulation of transcription factors underlies permissive bradyzoite differentiation in a natural isolate of Toxoplasma gondii
Source: mBio. 2024 Aug 16;15(9):e00641-24. doi: 10.1128/mbio.00641-24 (PMC11389365; doi:10.1128/mbio.00641-24)

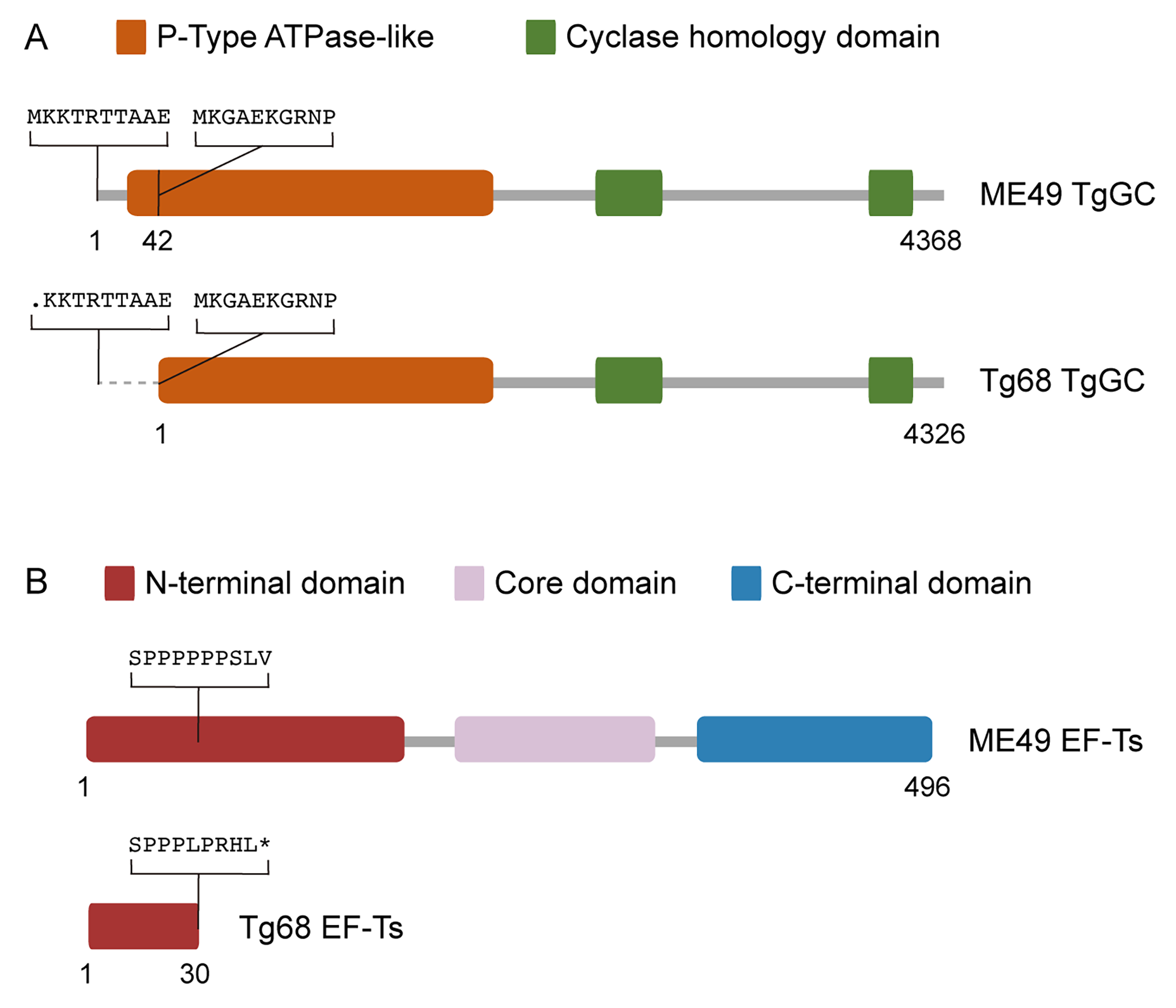

Supplement: Fig. S1 — Schematic showing gene variants in Tg68. [file mbio.00641-24-s0003.tif]

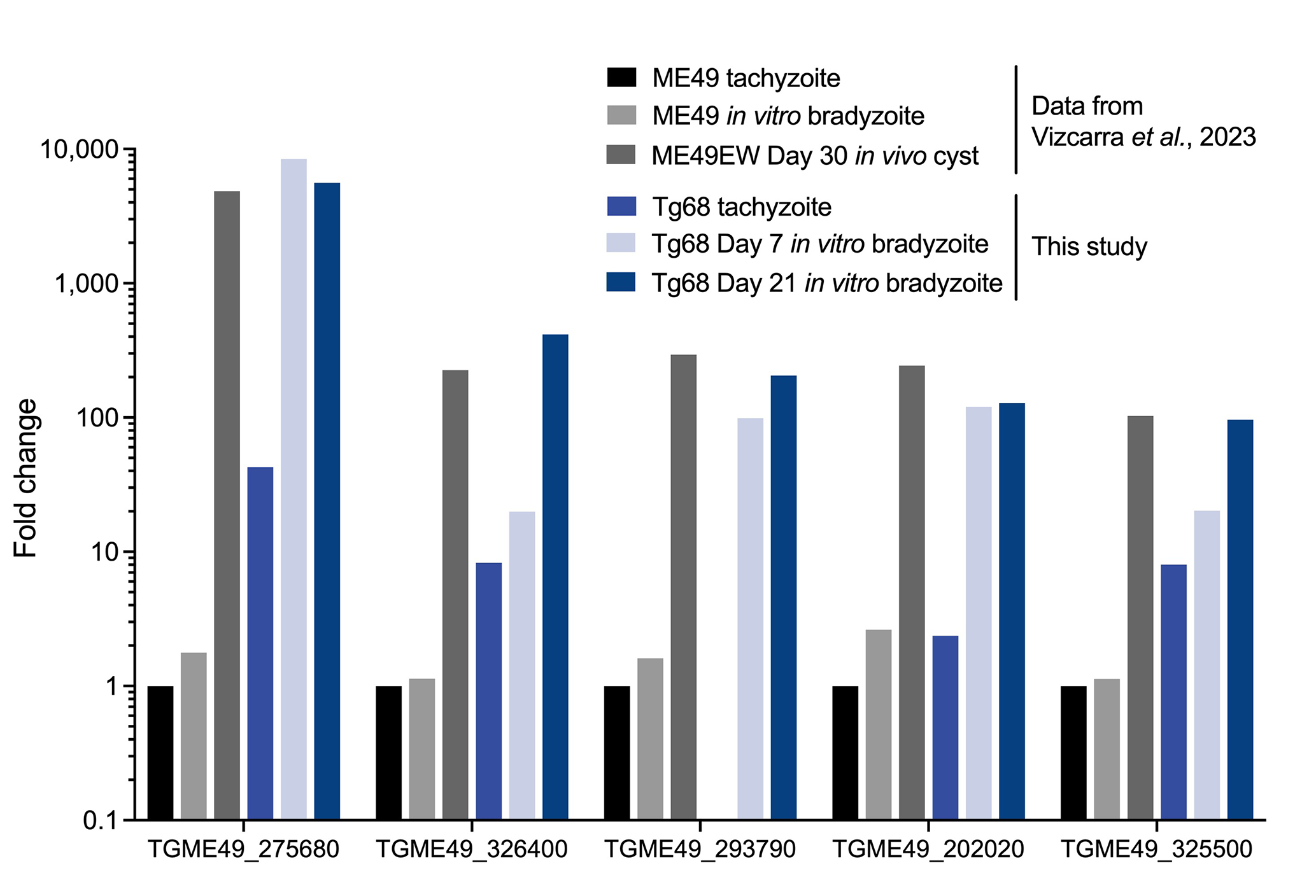

Supplement: Fig. S2 — Fold change of the in vivo bradyzoite-specific genes. [file mbio.00641-24-s0004.tif]

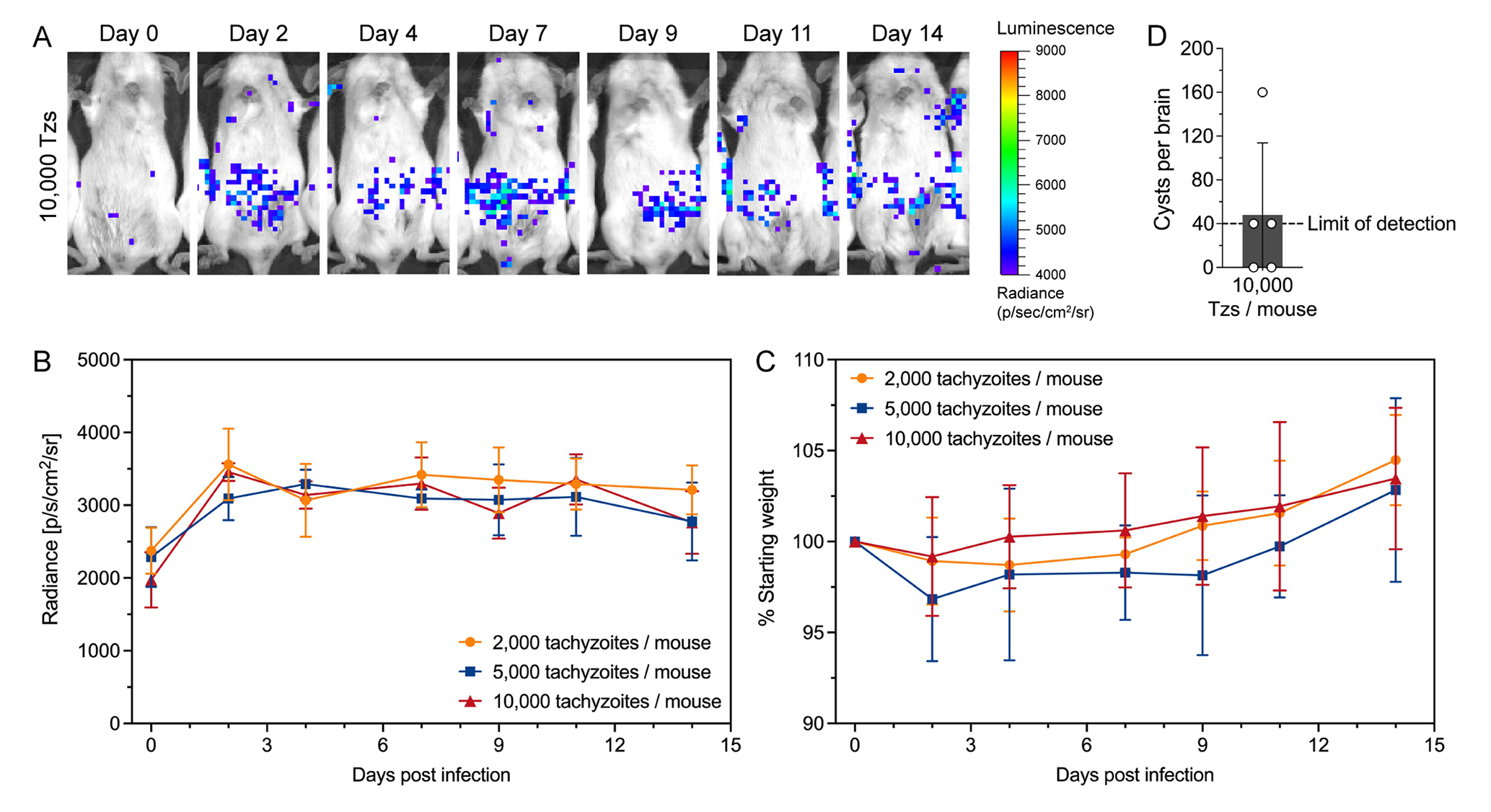

Supplement: Fig. S3 — Infection studies in CD-1 mice. [file mbio.00641-24-s0005.tif]

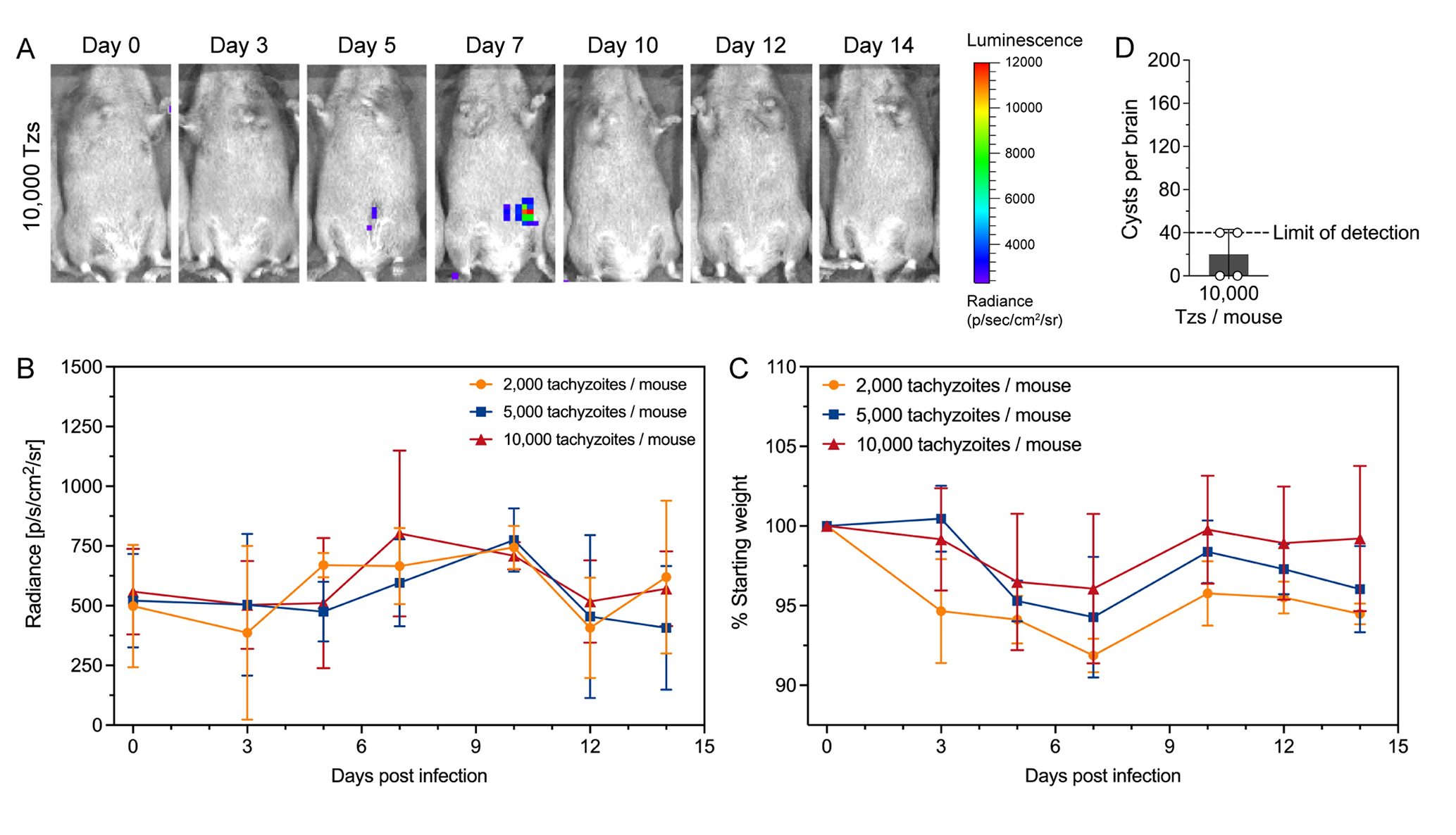

Supplement: Fig. S4 — Infection studies in CBA/CaJ mice. [file mbio.00641-24-s0006.tif]
