# Supplementary material for: Constitutive upregulation of transcription factors underlies permissive bradyzoite differentiation in a natural isolate of Toxoplasma gondii
Source: mBio. 2024 Aug 16;15(9):e00641-24. doi: 10.1128/mbio.00641-24 (PMC11389365; doi:10.1128/mbio.00641-24)
Supplement: Supplemental legends — Legends for Fig. S1 to S4. [file mbio.00641-24-s0007.docx]

**Figure S1**. Schematic showing the variants in (A) *TgGC* and (B) *EF-Ts* in Tg68, and their potential impact on the proteins.

**Figure S2**. Fold change of the *in vivo* bradyzoite-specific genes in ME49 *in vitro* bradyzoites, ME49EW day 30 *in vivo* cysts, Tg68 tachyzoites, Tg68 day 7 *in vitro* bradyzoites, and Tg68 day 21 *in vitro* bradyzoites, compared to ME49 tachyzoites.

**Figure S3** Female CD1 mice were infected intraperitoneally with 2,000, 5,000, and 10,000 Tg68 tachyzoites, monitored for parasite burden (A and B), weight loss (C), and cyst burden (D). (A and B) Bioluminescence imaging of Tg68 *TUB:FLuc,DHFR* infection was used to quantify parasite burden over the first 14 days of infection. (A) Representative images show radiance from a mouse infected with 10,000 Tg68 *TUB:FLuc,DHFR* tachyzoites. (B) Radiance (p/s/cm^2^/sr) in three groups of mice across different time points up to 14 days post infection is plotted as mean ± SD. (C) Percent weight of mice was calculated with the day before infection (Day 0) as 100% and is plotted as mean ± SD. (D) Number of cyst per brain in mice infected with 10,000 Tg68 *TUB:FLuc,DHFR* tachyzoites (Tzs) at day 31 post infection. Mean ± SD plotted for 5 animals.

**Figure S4** Female CBA/CaJ mice were infected intraperitoneally with 2,000, 5,000, and 10,000 Tg68 tachyzoites, monitored for parasite burden (A and B), weight loss (C), and cyst burden (D). (A and B) Bioluminescence imaging of Tg68 *TUB:FLuc,DHFR* infection was used to quantify parasite burden over the first 14 days of infection. (A) Representative images show radiance from a mouse infected with 10,000 Tg68 *TUB:FLuc,DHFR* tachyzoites. (B) Radiance (p/s/cm^2^/sr) in three groups of mice across different time points up to 14 days post infection is plotted as mean ± SD. (C) Percent weight of mice was calculated with the day before infection (Day 0) as 100% and is plotted as mean ± SD. (D) Number of cyst per brain in mice infected with 10,000 Tg68 *TUB:FLuc,DHFR* tachyzoites (Tzs) at day 35 post infection. Mean ± SD plotted for 4 animals.
